# Risk Factors Among HIV Seropositive Cases Attending a Tertiary Care Hospital of a North-Eastern State of India: A Hospital-Based Retrospective Cross-Sectional Study

**DOI:** 10.7759/cureus.80455

**Published:** 2025-03-12

**Authors:** Dina Raja, Harekrishna Nath, Rika Engtipi, Devyashree Medhi, Divya Daimari, Putul Mahanta

**Affiliations:** 1 Microbiology, Dhubri Medical College and Hospital, Dhubri, IND; 2 Forensic Medicine and Toxicology, Nalbari Medical College and Hospital, Nalbari, IND

**Keywords:** antiretroviral agents, cd4, influencing factor, injectable drug use, seropositivity

## Abstract

Introduction

Despite the relatively low HIV incidence in Assam, the region is at a critical juncture due to its strategic location as the gateway to north-eastern India and its proximity to three states with high HIV prevalence. This study, conducted from July 2022 to August 2023, aimed to assess the risk factors among HIV seropositive cases in Assam.

Methods

This hospital-based retrospective cross-sectional study included all HIV seropositive patients enrolled at the Integrated Counselling and Testing Centre (ICTC), Dhubri, Assam, India. The diagnosis was carried out following Strategy III of the National AIDS Control Organization (NACO), India guidelines, ensuring the thoroughness and reliability of the data. The difference in proportions was examined using a chi-square test with a p-value of less than 0.05 as significant. For identifying factors independently associated with the HIV transmission route, binary logistic regression was used. The data were analyzed using Statistical Package for the Social Sciences (SPSS) version 21 (IBM Corp., Armonk, NY).

Results

Out of 151 HIV seropositive patients, the majority, 133 (88.1%), were male. Most patients (95%, n=143) belonged to the young adult age group ranging from 15 to 49 years. A significant association between age of patients and gender was observed (p-value=0.002). HIV seropositivity was noted more among rural patients (n=99; 65.6%) and married female patients (p-value<0.001). The majority, 43.0% (n=65), of the patients were educated up to high school level. Injectable drug use was the leading mode of transmission (n=104; 68.9%). Route of HIV transmission was found to be significantly associated with age (p-value<0.001), gender (p-value<0.001), marital status (p-value<0.001), education (p-value<0.05), migration (p-value<0.001), and occupation (p-value<0.001).

Conclusion

The significant rise in new HIV infections, particularly among injectable drug users, underscores the urgent need for increased testing and awareness programs. These, along with better access to antiretroviral medication, rehabilitation programs, and stricter laws on illegal drug trafficking, could play a crucial role in controlling the spread of the disease.

## Introduction

Even though the HIV epidemic began more than 40 years ago, HIV still presents a threat to global public health [1]. Monitoring HIV transmission in a population is crucial for controlling and preventing the disease [2]. Chronic HIV infection is characterized by a steady loss of CD4+ T-cells and a disruption in CD4+ T-cell equilibrium, which eventually results in immune weakening and death [3].

HIV has claimed around 40.4 million (32.9-51.3 million) lives so far, making it a serious global public health concern [4]. The first HIV infections in India were discovered in 1986 among female sex workers in Chennai. As per the recent India HIV Estimation 2019 report, since the epidemic's peak in 2000, India's estimated adult HIV prevalence trend has been dropping and stabilizing in recent years. Yet, around 23.49 lakh people were living with HIV/AIDS (PLHIV) in India in 2019 [5]. North-eastern India constitutes both high and low HIV prevalent states, among which Mizoram had the highest estimated adult HIV prevalence (2.37%), followed by Nagaland (1.44%) and Manipur (1.15%). Assam is the gateway to north-eastern India and is surrounded by three states with high HIV prevalence. Thus, it is vulnerable to HIV transmission even if its HIV incidence is low in comparison to the rest of the nation [6]. A recent study from Assam documented HIV seropositivity as 4.68%, with males outnumbering females. The study also reported that the primary transmission route was the heterosexual route [7]. In India, unprotected homosexual and heterosexual behavior and injectable drug use are the primary contributors to the HIV epidemic, particularly among the high-risk groups [8]. People living with HIV/AIDS suffer from stigma associated with the virus, which has serious adverse effects on their psychological health and quality of life, and they often feel hesitant to get checked, treated and disclose their status due to fear of rejection or stereotypes [9].

Injectable drug users (IDUs), sex workers, and men who have sex with men (MSM) not only contribute towards a high burden of HIV, stigma, and prejudice but also increase the risk of blood-borne infections such as HIV, hepatitis B, and hepatitis C through high-risk behaviors such as sharing contaminated needles and syringes [10,11]. Spouses of IDUs are another category of usually monogamous women in India exposed to HIV and other infections because of their partners' high-risk behavior [11]. Sex work contributes to the HIV epidemic's propagation from the IDU population to sex workers and, ultimately, to the general public. The National AIDS Control Program places a strong emphasis on preventing new infections in high-risk populations. Targeted interventions are the most efficient way of halting the spread of HIV [12]. Designing tailored HIV prevention strategies requires understanding the sociodemographic and behavioral characteristics of the target population [12].

Determining the relative effects of behavioral risk variables may help direct HIV infection prevention efforts and aid in future HIV control planning in India. The current study aimed to assess the sociodemographic and behavioral characteristics of HIV seropositive cases attending a tertiary care hospital in Western Assam and thereby identifying the factors contributing to HIV infection.

## Materials and methods

The hospital-based retrospective study was conducted in the Department of Microbiology, Dhubri Medical College and Hospital, Dhubri, Assam, India, using data from all the HIV seropositive patients enrolled in the Integrated Counselling and Testing Centre (ICTC), Dhubri, from July 2022 to August 2023 after attaining approval from the Institutional Ethics Committee (DMCH/EC/2023/11) of Dhubri Medical College and Hospital and State Aids Control Society (ASACS), Assam.

After proper counselling and providing detailed information about the aims of the present study, informed consent was obtained from the participants. The participants were interviewed using a structured questionnaire to obtain information on age, sex, domicile, marital status, education, occupation, migration status, monthly income, and transmission route. The patients' socioeconomic status was documented as per the modified BG Prasad Classification [13], depending on the monthly per capita income. Strict confidentiality was maintained throughout the study period.

For HIV diagnosis, 2-3 mL of blood was collected aseptically in a clean, sterile tube from patients attending ICTC, Dhubri and tested for HIV antibody (HIV1/HIV2) in the serum following strategy III of the NACO, India guidelines [14]. Potential sources of bias, such as confounding and selection bias, were minimized by following exclusive diagnostic criteria for HIV and eliminating cases of exclusive criteria after proper history taking, clinical findings, and laboratory testing.

Sample size

The sample size was calculated using online Raosoft statistical software and was estimated at n=151, considering a seropositivity rate of 11% [6], a confidence level of 95%, and a margin of error of 5%. The power analysis using G*Power software revealed that the sample size n=151 is sufficient to detect meaningful associations using chi-square contingency tables with 80% power and an effect size of 0.3.

Statistical analysis

The data were entered into MS Excel and analyzed using Statistical Package for the Social Sciences (SPSS) version 21 (IBM Corp., Armonk, NY). The mean and standard deviation were used to present the continuous variables. In contrast, percentages and frequencies were used to present categorical variables. The Kolmogorov-Smirnov test was used to examine the normality of a continuous variable's distribution. The proportional differences were examined for statistical significance using a non-parametric chi-square test. Univariate and multivariate binary logistic regression models with backward elimination were used to identify independent factors associated with the HIV transmission route. A p-value of less than 0.05 was deemed statistically significant.

## Results

A total of 151 patients seropositive for HIV infection were included in the study, of whom 133 (88.1%) patients were male and 18 (11.9%) were female. The patients' mean age (±standard deviation) was 26.97 (±8.94) years. Approximately 94.7% (n=143) of the seropositive patients belonged to the age group of 15-49 years, with the highest number of cases found in the age group of 15-24 years (n=67; 44.3%), followed by the age group of 25-34 years (n=60; 39.7%) and 35-49 years (n=16; 10.6%). A significant association between the age of patients and gender was observed (p-value=0.002), with 84% (n=117) of the male patients being in the age group of 15-34 years. Higher incidences of HIV seropositive patients were from rural areas (n=99; 65.6%). High HIV seropositivity was significantly noted among married female patients (p-value<0.001), while the HIV seropositivity status was almost similar among married and unmarried male patients. The majority, 43.0% (n=65), of the patients were educated up to high school level. Almost 10.6% (n=16) of patients were migrants (Table 1).

**Table 1:** Sociodemographic characteristics of the HIV seropositive cases The data are presented as frequency (n) and percentage (%), with a total sample size of n=151. *A p-value of <0.05 was considered statistically significant for the chi-square test

Variables	Categories	Sex		Chi-square value	p-Value
		Male (n=133)	Female (n=18)		
Age	0-14 years	2 (1.5%)	1 (5.6%)	17.06	0.002*
	15-24 years	61 (45.9%)	6 (33.3%)		
	25-34 years	56 (42.1%)	4 (22.2%)		
	35-49 years	12 (9.0%)	4 (22.2%)		
	≥50 years	2 (1.5%)	3 (16.7%)		
Domicile	Urban	49 (36.8%)	3 (16.7%)	2.85	0.09
	Rural	84 (63.2%)	15 (83.3%)		
Marital status	Unmarried	65 (48.9%)	2 (11.1%)	28.58	<0.001*
	Married	68 (51.1%)	13 (72.2%)		
	Widowed/separated	0 (0.0%)	3 (16.7%)		
Education	Illiterate	30 (22.6%)	6 (33.3%)	3.26	0.51
	Primary	14 (10.5%)	2 (11.1%)		
	High school	58 (43.6%)	7 (38.9%)		
	Intermediate	24 (18.0%)	1 (5.6%)		
	Graduation and above	7 (5.3%)	2 (11.1%)		
Migrants	No	119 (89.5%)	16 (88.9%)	0.005	0.94
	Yes	14 (10.5%)	2 (11.1%)		

**Figure 1:**
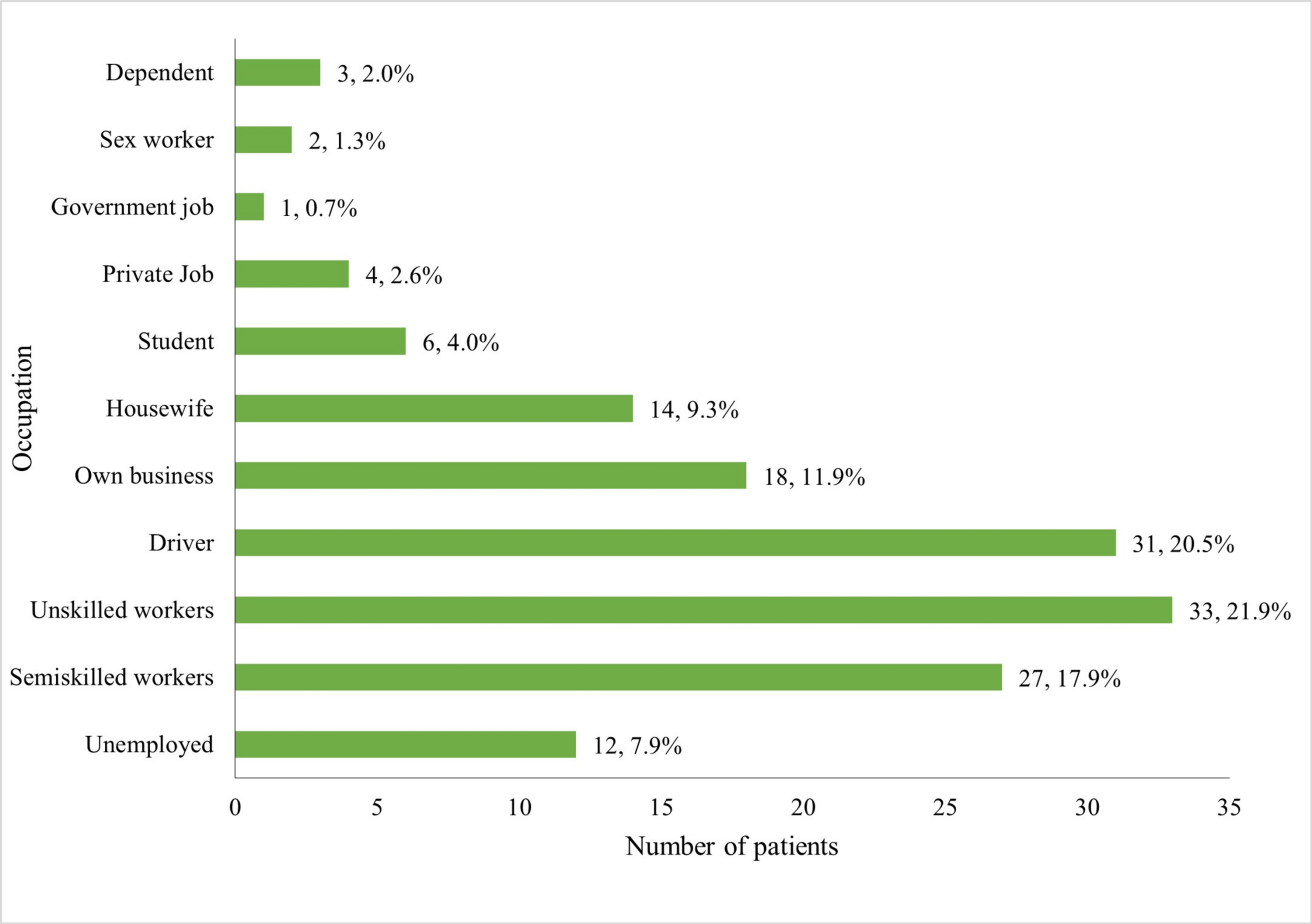
Occupation-wise distribution of HIV seropositive cases The data are presented as frequency (n) and percentage (%), with a total sample of n=151

Out of the 151 patients, 21.9% (n=33) were unskilled laborers, 17.9% (n=27) were engaged in semiskilled work, and 7.9% (n=12) were unemployed. Long or short-distance drivers constituted 20.5% (n=31) of the patients. Around 11.9% (n=18) of patients had their own business, 9.3% (n=14) were homemakers, and 4.0% (n=6) were students (Figure 1).

**Figure 2:**
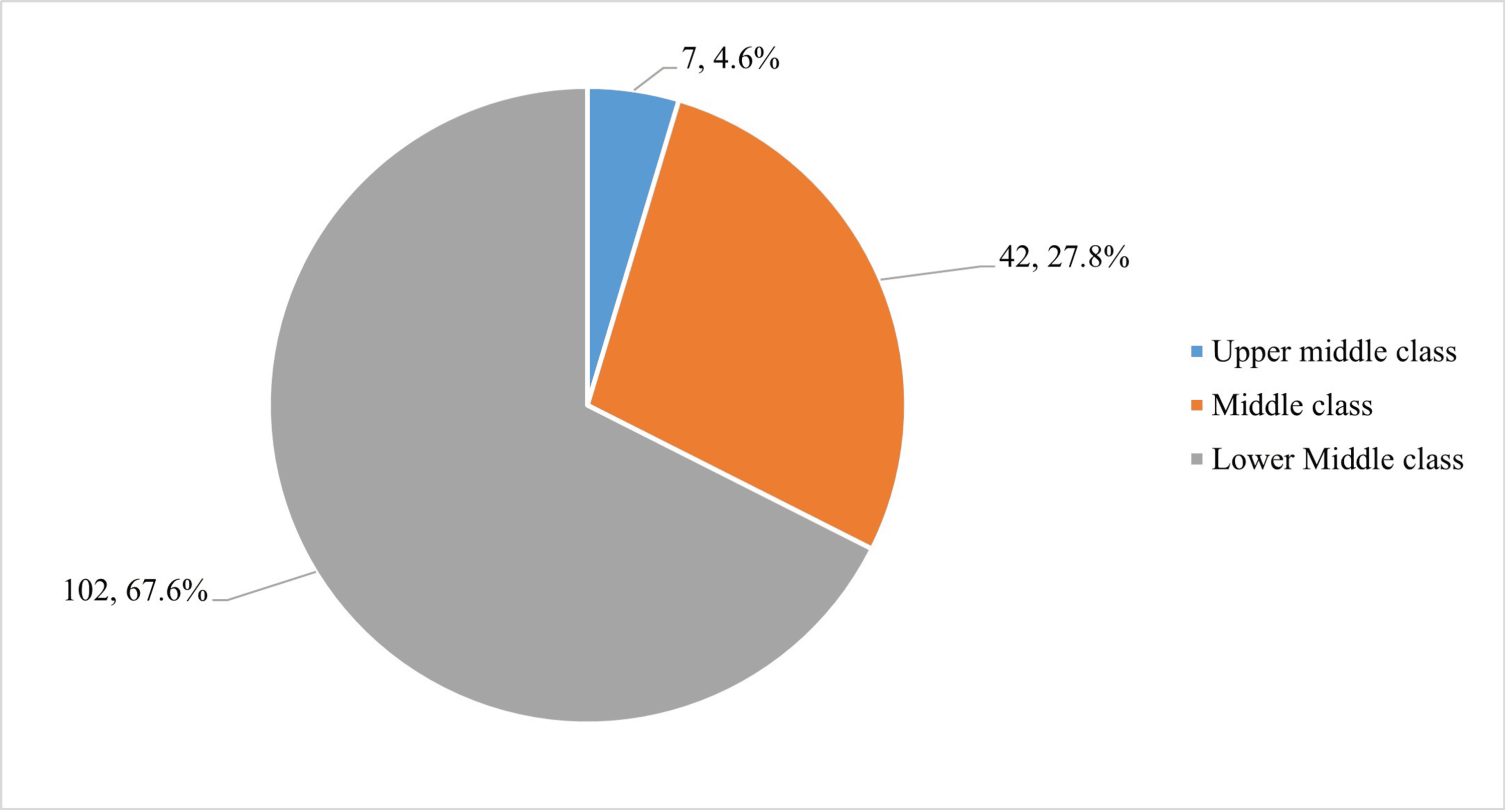
Socioeconomic status of HIV seropositive cases The data are presented as frequency (n) and percentage (%), with a total sample size of n=151

Almost two-thirds of the patients (n=102; 67.6%) were from lower middle-class economic status, 27.8% (n= 42) belonged to a middle class, and only 7 (4.6%) out of 151 patients belonged to the upper middle class as per the modified BG Prasad classification of socioeconomic status (Figure 2).

While observing the mode of transmission, we have found that the highest number of HIV patients (n=104; 68.9%) were IDUs; heterosexual activity with one or many partners was noted in 43 (28.5%) patients. Vertical route transmission was observed in three (1.98%) patients, all below 15 years of age. Homosexual route of transmission was found in only one (0.7%) male patient (Table 2).

**Table 2:** Mode of transmission of HIV infection The data are presented as frequency (n) and percentage (%), with a total sample of n=151

Mode of transmission	Number of patients	Percentage
Injectable drug use	104	68.90%
Parent to child	3	2.00%
Heterosexual intercourse	43	28.50%
Homosexual intercourse	1	0.70%

As shown in Table 3, the association between the route of transmission and sociodemographic variables was further investigated among the 148 HIV seropositive cases, excluding the three cases of vertical route transmission. A significant association between the age of the patient and the route of HIV transmission was noted (p-value<0.001), with the majority of the IDUs belonging to the age group of 15-34 years (n=100/104, 96.1%). It was also observed that HIV infection among IDUs is increasing (p-value <0.001) and predominantly in males (n=104; 100%). Sexual transmission of HIV was substantially noted among married patients (p-value<0.001). More than 80.0% (n=85/104) of the IDUs were literate (p-value<0.05), and none of the migrant patients were IDUs (p-value<0.001). A highly significant association was noted between the route of HIV transmission and the occupation of the patients (p-value<0.001). Injectable drug use was observed to be the route of transmission mostly among unskilled laborers (n=25/104; 24.0%), drivers (n=25/104; 24.0%), and semiskilled workers (n=21/104; 20.2%). On the other hand, sexual transmission of HIV was mainly noted among homemakers (n=14/44; 31.8%), followed by unskilled laborers (n=8/44; 18.2%).

**Table 3:** Association of sociodemographic variables and route of transmission of HIV The data are presented as frequency (n) and percentage (%), with a total sample size of n=148 *A p-value of <0.05 was considered statistically significant for the chi-square test. #Sexual transmission route comprised both homosexual and heterosexual routes of transmission

Variable	Categories	Transmission route		Chi-square value	p-Value
		Sex^# ^(n=44)	IDU (n=104)		
Age group	15-24 years	10 (22.7%)	57 (54.8%)	34.60	<0.001*
	25-34 years	17 (38.6%)	43 (41.3%)		
	35-49 years	12 (27.3%)	4 (3.8%)		
	≥50 years	5 (11.4%)	0 (0.0%)		
Gender	Male	27 (61.4%)	104 (100.0%)	45.39	<0.001*
	Female	17 (38.6%)	0 (0.0%)		
Domicile	Urban	14 (31.8%)	38 (36.5%)	0.30	0.58
	Rural	30 (68.2%)	66 (63.5%)		
Marital status	Unmarried	8 (18.2%)	56 (53.8%)	20.89	<0.001*
	Married	33 (75.0%)	48 (46.2%)		
	Widowed/separated	3 (6.8%)	0		
Education level	Illiterate	15 (34.1%)	19 (18.3%)	4.37	0.04*
	Literate	29 (65.9%)	85 (81.7%)		
Migrant	No	28 (63.6%)	104 (100.0%)	42.40	<0.001*
	Yes	16 (36.4%)	0 (0.0%)		
Occupation	Unemployed	1 (2.3%)	11 (10.6%)	48.23	<0.001*
	Semiskilled worker	6 (13.6%)	21 (20.2%)		
	Unskilled worker	8 (18.2%)	25 (24.0%)		
	Own business	5 (11.4%)	13 (12.5%)		
	Student	0 (0.0%)	6 (5.8%)		
	Housewife	14 (31.8%)	0 (0.0%)		
	Driver	6 (13.6%)	25 (24.0%)		
	Private job	1 (2.3%)	3 (2.9%)		
	Government job	1 (2.3%)	0 (0.0%)		
	Sex worker	2 (4.5%)	0 (0.0%)		

The univariate logistic regression analysis revealed that those aged 35 years and below had a 15 times higher chance of HIV transmission through injectable drug use (OR=15.7, 95% CI: 4.9-50.7). Also, the chances of transmission of HIV through injectable drug use were five-fold higher among unmarried individuals (OR=5.2, 95% CI: 2.2-12.3). Almost two times higher odds of HIV transmission through injectable drug use were observed among literate participants (OR=2.3, 95% CI: 1.0-5.1) as compared to illiterate individuals. As compared to housewives and those belonging to other professions, unemployed persons (OR=11.5, 95% CI: 1.4-96.7), those involved in semiskilled (OR=3.7, 95% CI: 1.2-10.8) and unskilled workers (OR=3.3, 95% CI: 1.2-8.8), and drivers (OR=4.3, 95% CI: 1.5-12.6) had significantly higher odds of HIV transmission through injectable drug use. The data were further analyzed using multivariate logistic regression with the backwards Wald elimination method to adjust potential confounding effects. The multivariate logistic regression analysis revealed that age of 35 years and below (OR=15.6, 95% CI: 3.6-67.3), unmarried individuals (OR=4.0, 95% CI: 1.5-11.0), individuals engaged in semiskilled (OR=4.1, 95% CI: 1.2-14.5) and unskilled works (OR=4.7, 95% CI: 1.4-15.5), and drivers (OR=13.7, 95% CI: 3.1-61.4) had significantly higher chances of HIV transmission through injectable drug use (Table 4).

**Table 4:** Logistic regression analysis for association of sociodemographic variables and route of transmission of HIV IDU, injectable drug use; OR, odds ratio; ref, reference category; 95% CI, 95% confidence interval lower and upper bound #Sexual transmission route comprised both homosexual and heterosexual routes of transmission

Variable	Categories	Transmission route		Crude OR	95% CI	Adjusted OR	95% CI
		Sex^# ^(n=44)	IDU (n=104)				
Age group	35 years and above	17 (38.6%)	4 (3.8%)	1 (ref)	-	1 (ref)	-
	15-35 years	27 (61.4%)	100 (96.2%)	15.7	4.9-50.7	15.6	3.6-67.3
Marital status	Married/widowed/separated (n=84)	36 (81.8%)	48 (46.2%)	1 (ref)	-	1 (ref)	-
	Unmarried (n=64)	8 (18.2%)	56 (53.8%)	5.2	2.2-12.3	4.0	1.5-11.0
Education level	Illiterate (n=34)	15 (34.1%)	19 (18.3%)	1 (ref)	-	1 (ref)	
	Literate (n=114)	29 (65.9%)	85 (81.7%)	2.3	1.0-5.1		
Occupation	Housewife/others (n=45)	23 (52.3%)	22 (21.2%)	1 (ref)	-	1 (ref)	-
	Semiskilled worker (n=27)	6 (13.6%)	21 (20.2%)	3.7	1.2-10.8	4.1	1.2-14.5
	Unskilled worker (n=33)	8 (18.2%)	25 (24.0%)	3.3	1.2-8.8	4.7	1.4-15.5
	Driver (n=31)	6 (13.6%)	25 (24.0%)	4.3	1.5-12.6	13.7	3.1-61.4
	Unemployed (n=12)	1 (2.3%)	11 (10.6%)	11.5	1.4-96.7	5.7	0.6-51.4

## Discussion

In the past 20 years, India's National AIDS Control Programme has developed and grown to offer HIV prevention, testing, and treatment services nationwide. All strategic elements have experienced consistent scaling up, which has prevented and even reversed the epidemic's spread and guaranteed a significant drop in the yearly number of AIDS-related fatalities. The HIV scenario in India is exceptionally diverse, mainly concentrating on high-risk populations and specific regions across the nation [15]. Although the advent of COVID-19 has not significantly influenced government efforts to contain the HIV/AIDS epidemic, an increase in HIV rates was observed in a few areas of India, including Assam [16]. Region-specific data on sociodemographic and behavioral patterns of people living with HIV might help understand the effects of those on HIV transmission.

In the present study, a total of 151 patients were found seropositive for HIV infection during the study period, where male participants (n=133, 88.1%) outnumbered female participants (18, 11.9 %). Our findings are in contrast to a recent study in which females were observed to carry a proportionately larger burden of HIV incidence in India despite a narrower gender gap in HIV incidence [2]. Almost 95% (n=143) of the patients in the current study belonged to the age group of 15-49 years. A significant association between the age of patients and gender (p-value=0.002) was noted, with the majority, 88% (n=117), of male patients belonging to the age group of 15-34 years. Another study reported that women between 15 and 24 years of age are twice as likely as young men to be infected with HIV globally and that young women alone account for almost 25% of all new HIV infections [17].

In the current study, HIV seropositive females were mainly married (p-value<0.001). Negotiating the use of condoms and other safe-sex practices in marriage and other intimate relationships is very difficult for women who experience intimate partner violence [18]. Also, due to their partners' high-risk behavior, spouses of IDUs are another group of typically monogamous Indian women who are at risk for HIV and other diseases [11].

The present study shows a high frequency of HIV infection (n=104; 68.9%) among IDUs, followed by heterosexual transmission (n=43; 28.5%) through one or many partners. Vertical route transmission was noted in three (2.0%) patients below 15 years of age. Homosexual (MSM) transmission was reported in only one (0.7%) patient. Excluding the three cases of vertical route transmission from the analysis, the relationship between the route of transmission and sociodemographic factors was further examined for the 148 adult HIV-positive cases. A significant association between the age of the patient and the route of HIV transmission was noted (p-value<0.001), with the majority of the IDUs belonging to the age group of 15-34 years (n=100/104, 96.1%). The age bracket of 15-34 years showed an alarming 15-fold high risk of HIV transmission via injectable drug use (OR=15.7, 95% CI: 4.9-50.7). The findings are in agreement with another recent study [19]. Increasing HIV infection among male IDUs (p-value <0.001) in the study region is alarming. It necessitates the strengthening of IDU-related awareness and interventions along with stricter law enforcement against illicit drug use. More than 80.0% (n=85/104) of the IDUs were literate (p-value<0.05). The high prevalence of HIV among literate IDUs indicates the lack of health literacy among the substance users in the region [20]. Targeted intervention programs have proved to be helpful in the behavioral change of IDUs by the use of new needles and syringes [18]. Sexual transmission of HIV was substantially noted among married patients (p-value<0.01). Lack of awareness about healthy sexual behavior and intimate partner violence are primarily attributable to the transmission of HIV among married couples. A recent study documented the use of condoms in only 18% of married heterosexual HIV sero-discordant couples [21].

A highly significant association was noted between the route of HIV transmission and the occupation of the patients (p-value<0.01). Sexual transmission of HIV was mainly noted among homemakers (n=14/44; 31.8%). Injectable drug use was observed to be the route of transmission mostly among unskilled laborers (n=25/104; 24.0%), drivers (n=25/104; 24.0%), and semiskilled workers (n=21/104; 20.2%). The multivariate logistic regression model revealed that HIV transmission through injectable drug use was significantly more likely among unmarried people (OR=4.0, 95% CI: 1.5-11.0), people engaged in semiskilled (OR=4.1, 95% CI: 1.2-14.5) and unskilled jobs (OR=4.7, 95% CI: 1.4-15.5), and drivers (OR=13.7, 95% CI: 3.1-61.4). Similar to our findings, a recent review documented educational status and employment as a significant factor of HIV transmission among IDUs [22].

The study also found that there was an increase in HIV seropositivity in IDUs over the one-year study period. This could be attributed to more people visiting ICTC, Dhubri, for HIV testing since its initiation two years ago. Also, the lack of information on HIV-positive female IDUs does not rule out the possibility of hidden transmission among female IDUs, which might be left unscreened [22].

Geographical proximity, border sharing with Bangladesh, lack of resources, sociopolitical issues, low educational attainment, lack of addiction treatment, and the recent use of unsterilized injection equipment have all significantly impacted the spread of HIV among IDUs and the failure of HIV/AIDS prevention programs in the study region. Additionally, stigma, discrimination, and misinformation all play a significant role in the spread of HIV because they hinder the proper dissemination of pertinent information and the implementation of efficient preventative measures.

Limitations

The findings of the study will aid researchers in their understanding of the variables associated with HIV infection in high-prevalence regions. However, the present study is limited to one year and includes data from only one ICTC center. Also, the study aims to assess the sociodemographic and behavioral characteristics of the HIV seropositive cases associated with HIV transmission. Further investigations with longer duration and larger sample sizes, including both behavioral and clinical factors such as co-infections, drug history, and drug adherence, might help in identifying the key factors and other confounders associated with increased HIV transmission in the region. A more extensive multicentric study including data from other ICTCs of the state could highlight the HIV spectrum of the region more prominently.

## Conclusions

HIV seropositivity was higher among the adult population and males. Injectable drug use and heterosexual mode of transmission were the most prominent modes of HIV transmission. Age, gender, education status, marital status, and occupation were significantly associated with the route of HIV transmission among adult patients.

Drug injecting-related HIV programs related to comprehensive harm reduction, including clean needle and syringe exchange and opioid substitution treatment, should be extended for underprivileged and rural areas promptly. Preventive measures are needed, including psychological counselling and targeted intervention for behavioral change in high-risk groups. Early screening for IDUs for the detection of HIV and psychological counselling for antiretroviral therapy might guarantee low viremia in individuals with HIV, thereby providing them with the same level of health and life expectancy as their counterparts without HIV.

## References

[1] Fauci AS, Lane HC (2020). Four decades of HIV/AIDS - much accomplished, much to do. N Engl J Med.

[2] Shri N, Bhattacharyya K, Dhamnetiya D, Singh M, Jha RP, Patel P (2023). Long-term trends of HIV/AIDS incidence in India: an application of joinpoint and age-period-cohort analyses: a gendered perspective. Front Public Health.

[3] Vidya Vijayan KK, Karthigeyan KP, Tripathi SP, Hanna LE (2017). Pathophysiology of CD4+ T-cell depletion in HIV-1 and HIV-2 infections. Front Immunol.

[4] (2025). Epidemiological fact sheet. https://cdn.who.int/media/docs/default-source/hq-hiv-hepatitis-and-stis-library/j0294-who-hiv-epi-factsheet-v7.pdf.

[5] (2025). India HIV Estimates 2019: Report. https://www.aidsdatahub.org/sites/default/files/resource/india-hiv-estimates-2019.pdf.

[6] (2025). Present HIV/AIDS Scenario. https://asacs.assam.gov.in/information-services/present-hivaids-scenario.

[7] Hazarika NK, Alam ST, Sarmah A, Bhagawati A (2016). A retrospective study on the prevalence of HIV among patients attending a tertiary care hospital of northeast India. World J AIDS.

[8] Bachani D, Sogarwal R (2010). National Response to HIV/AIDS in India. Indian J Community Med.

[9] Tadesse G, Rtbey G, Andualem F (2024). HIV-related perceived stigma and internalized stigma among people living with HIV/AIDS in Africa: a systematic review and meta-analysis. PLoS One.

[10] Risher K, Mayer KH, Beyrer C (2015). HIV treatment cascade in MSM, people who inject drugs, and sex workers. Curr Opin HIV AIDS.

[11] Solomon SS, Srikrishnan AK, Celentano DD (2011). The intersection between sex and drugs: a cross-sectional study among the spouses of injection drug users in Chennai, India. BMC Public Health.

[12] Sinha AK, Samantaray A, Panda PS, Shinkar SV (2022). Status of socio-demographic and behavioral profile of younger and older HIV high risk groups in Chhattisgarh. J Family Med Prim Care.

[13] (2022). National Guidelines for HIV Testing. https://www.naco.gov.in/sites/default/files/National_Guidelines_for_HIV_Testing_21Apr2016.pdf.

[14] K J A, Mishra A, Borle AL (2024). Updated BG Prasad scale for socioeconomic status classification for the year 2024. Indian J Pediatr.

[15] Tanwar S, Rewari BB, Rao CD, Seguy N (2016). India's HIV programme: successes and challenges. J Virus Erad.

[16] Varshney K, Mustafa AD (2024). Trends in HIV incidence and mortality across Bharat (India) after the emergence of COVID-19. Int J STD AIDS.

[17] Richardson ET, Collins SE, Kung T (2014). Gender inequality and HIV transmission: a global analysis. J Int AIDS Soc.

[18] Shri N, Muhammad T (2021). Association of intimate partner violence and other risk factors with HIV infection among married women in India: evidence from National Family Health Survey 2015-16. BMC Public Health.

[19] Sahu D, Ranjan V, Chandra N, Nair S, Kumar A, Arumugam E, Rao MV (2022). Analysis of a targeted intervention programme on the risk behaviours of injecting drug users in India: evidence from the National Integrated Biological and Behavioural Surveillance Survey. J Prev Med Public Health.

[20] Rolova G, Gavurova B, Petruzelka B (2021). Health literacy, self-perceived health, and substance use behavior among young people with alcohol and substance use disorders. Int J Environ Res Public Health.

[21] Beri LV, Shelke PS, Acharya SM (2021). Sexual behavior and contraceptive practices: Study among married heterosexual HIV serodiscordant couples of reproductive age group attending anti-retroviral therapy centre at a Tertiary care hospital in Mumbai, India. J Family Med Prim Care.

[22] Pachuau LN, Tannous C, Dhami MV, Agho KE (2022). HIV among people who inject drugs in India: a systematic review. BMC Public Health.

